# Biomechanical Analysis of the Field Hockey Sweep Skill Using Inertial Measurement Units

**DOI:** 10.3390/s26103095

**Published:** 2026-05-14

**Authors:** Hillary Cox, Rachel V. Vitali

**Affiliations:** Department of Mechanical Engineering, University of Iowa, Iowa City, IA 52242, USA; hillary-cox@uiowa.edu

**Keywords:** wearable sensors, inertial measurement unit, field hockey, sport performance

## Abstract

Wearable sensors like inertial measurement units (IMUs) can quantify sport technique in natural settings, yet field hockey-specific skill analyses remain limited. This exploratory study investigated how relative foot placement, stick orientation, and lower body kinematics at impact relate to performance of the field hockey sweep skill. Eight experienced female participants performed sweeps under three foot positions relative to the ball (in front, in line, and behind) while IMUs recorded body segment and stick motion. Sweep performance was characterized by accuracy, bounciness, and ball speed. Placing the foot in front of the ball was associated with reduced ball speed and a trend toward lower accuracy relative to the in-line reference, whereas placing the foot behind the ball did not differ from in line on any outcome. Stick roll at impact emerged as a consistent trial-level predictor, with a more open face associated with a greater likelihood of a bouncy sweep and slightly increasing ball speed. Stick pitch and lower limb joint angles were not significant within-participant predictors. However, between-participant analyses indicated that larger knee angles and smaller hip angles were associated with greater accuracy, while smaller average pitch was associated with faster ball speed. Together, these findings indicate that some aspects of sweep performance are amenable to immediate technique adjustments whereas others reflect stable individual movement tendencies. These findings provide a foundation for future work on offering evidence-based guidance for technique refinement and potential implications for injury risk reduction.

## 1. Introduction

Participation in sports provides numerous physical, psychological, and social benefits at all stages of life [1]. Engaging in sports, particularly from a young age, is associated with higher levels of lifelong physical activity and health benefits [2] in addition to psychosocial development like social skills, emotional regulation, and self-esteem [3,4]. However, sports participation can also entail significant risks. For example, performance pressure has been linked to poor mental health outcomes, especially among high-level athletes [5], and reports of physical, psychological, and sexual abuse have raised serious concerns given the high prevalence of youth participation in the U.S. [6,7]. Additional potential harms include the development of eating disorders [8] and injury risk across all levels of participation (i.e., from youth to professional sport) [9,10].

Wearable sensor technologies have been increasingly adopted in sports to mitigate some of these key drawbacks associated with participation, particularly injury risk and sustained pressure to perform at an elite level [11]. By enabling continuous monitoring of both internal (e.g., heart rate, perceived exertion, and physiological stress) and external (e.g., distance covered, accelerations, and impacts) training loads, wearable sensors provide objective insights into athlete fatigue, workload accumulation, and recovery status, which are strongly linked to injury incidence [12,13]. Inertial measurement units (IMUs), global navigation satellite systems (GNSSs), and physiological sensors (e.g., electromyography and peripheral oxygen saturation) allow practitioners to identify maladaptive movement patterns, excessive mechanical loading, and workload spikes that increase injury risk [14,15]. Beyond physical injury prevention, wearable sensor-derived feedback reduces performance pressure by facilitating data-informed decision making, which shifts evaluation from subjective judgment toward athlete-centered metrics that can guide training intensity, rehabilitation progression, and return-to-play decisions [16,17]. Recent advances further demonstrate that machine learning applied to IMU-derived signals can help identify maladaptive movement patterns and fatigue onset with relatively high accuracy, which supports the development of real-time feedback systems for both injury prevention and technique refinement in training environments [18]. Consequently, wearable sensors play a dual role in modern sport; namely, protecting athlete health while fostering sustainable performance development in ecologically valid, real world environments.

Of particular interest to this work is the sport of field hockey, an internationally popular recreational activity that has a relatively high rate of injury among its participants. Up to 75% of field hockey players sustain at least one injury during a game or practice in a typical season [19], which is similar to other team sports like basketball and soccer [20]. While many injuries are associated with impacts from the ball or stick [21], non contact related injuries and pain are also common [22,23]. As a result, field hockey studies involving wearable sensors have often focused on injury risk and injury prevention, though there is limited evidence showing field hockey specific interventions can effectively prevent injuries [24]. For example, Wilmes et al. [25] demonstrated that IMUs can quantify field hockey-specific biomechanical loads (e.g., time spent in a lunge position and hip load) that are not well represented by traditional distance-based metrics. While these metrics offer valuable insight into sport-specific loading and potential contributors to injury risk, the study stops short of evaluating whether such information can meaningfully reduce injury incidence, reflecting a broader gap in the current literature.

Subsequent work has expanded the scope of IMU-based monitoring in field hockey. For instance, Wilmes et al. [26] examined a range of metrics during small-sided field hockey games using IMUs attached to the pelvis and thighs. Subsequent analysis revealed that different game formats produce meaningfully distinct biomechanical demands, such as loads originating from rapid acceleration and time spent in physically demanding postures. Complementing this work, a 2024 systematic review of wearable devices deployed in field hockey identified 6 distinct use cases for these technologies: (1) performance monitoring, (2) technical skill analysis, (3) game strategy, (4) injury prevention, (5) workload management, and (6) physiological measurement [27]. Of the 3400 screened titles, only 6 of the 23 eligible studies addressed technical skill analysis, and none employed IMUs as the primary tool for linking skill-specific kinematics to performance outcomes. At the level of individual technical skills, IMUs have also been used to characterize joint level demands. For example, a 2024 observational study identified differences in hip and ankle range of motion during the drag flick that distinguished higher- from lower-level players [28]. Together, these studies underscore that while wearable sensor technology is rapidly maturing in field hockey, its application to specific technical skills, particularly biomechanical analysis tied to performance outcomes, remains a somewhat underexplored area of research.

Coaching cues often emphasize specific technique recommendations that are assumed to enhance performance, yet these instructions may also influence (non contact) injury risk. For example, field hockey players are frequently encouraged to adopt very low playing positions when executing certain skills; however, there is limited empirical evidence supporting improved performance from these cues, and such postures may contribute to injury etiology. Despite the potential implications for both performance and injury risk, the biomechanical underpinnings of technical skills in field hockey remain relatively understudied, leaving uncertainty about the factors that truly contribute to successful skill execution. Multiple studies have explored the kinematics of the field hockey hit, including the planarity of the stick face during the hit skill [29], how the execution of the hit skill differs between defenders and attackers/midfielders [30], and comparisons of the upper limb kinematics of short grip and classic drives [31]. While the kinematics of certain field hockey skills are relatively understood, limited research examines the connection between common coaching cues and the kinematic determinants of successful performance. For example, elite players performing a drag flick skill typically exhibit a wide stance, a pronounced whipping action, and rapid sequential movement of the pelvis, upper trunk, and stick [32]. However, players that specialize in that explosive whipping movement generated by rotating the pelvis and trunk in a low posture [33] experience significantly higher rates of hip and lower back injury [34]. A 2023 systematic review of the drag flick skill identified 16 eligible studies encompassing 142 drag-flickers and found that while numerous individual kinematic parameters were associated with drag flick performance, the overall body of evidence remains limited in quantity and methodological quality [35]. As such, the authors called for future research to develop a clearer biomechanical characterization of this relatively complex motor skill. However, to date, skill-specific biomechanical research in field hockey has largely concentrated on the aforementioned drag flick and hit skills, while the sweep skill remains understudied. Although motion capture analyses have been used to examine injury risk related to ball position, there is limited evidence addressing coaching cues or the kinematic factors underlying successful sweep performance [36].

The purpose of this exploratory study is to investigate the biomechanical factors that influence performance of the field hockey sweep skill. To collect data in as realistic an environment as possible, IMUs were employed to track motion. Despite the common pieces of coaching advice given while performing this skill, it is not well understood what factors contribute to a successful sweep, which is defined in many contexts as fast, accurate, and flat (i.e., not bouncy). Some advanced field hockey strategy requires a sweep to be purposefully hit into the air, but the objective of most sweeps is to be flat. To ensure a range of performances, participants completed the sweeps with different foot positions with respect to the ball placement. It is hypothesized that relative foot position, stick orientation, and joint angles at impact with the ball will influence the performance of the sweep skill.

## 2. Methods

### 2.1. Participants

Participants were eligible to participate if they were over the age of 18, self-identified as female, had competed in field hockey within the past four years, and were familiar with the sweep skill. Additional inclusion required self-reporting of no perceived or diagnosed conditions that could limit participation, including: (1) muscle injuries that limit physical activity, (2) recent fractures within the past 6 months, (3) vestibular disorders that affect balance, (4) currently participating in rehabilitation after surgery, and (5) heart conditions that require recurring follow-up. In the United States, field hockey is primarily regarded as a women’s sport, which is why only female participants were recruited. Demographic information on participant height, age, nationality, team membership, and sweep experience was collected for each participant at the beginning of the data collection. A sample of 8 female participants (19.9 ± 1.6 years; 171 ± 6 cm) with extensive experience performing the field hockey sweep skill was recruited for this study. Five participants grew up in the United States, while the remaining three participants grew up in another country. Only one participant self-identified as someone who regularly performed a sweep during penalty corner plays. Participants used their own field hockey sticks for the data collection. All participants provided informed consent to be part of this study, and all study activities were approved by the University of Iowa Institutional Review Board under ID #202401659.

### 2.2. Experimental Procedure

**Figure 1:**
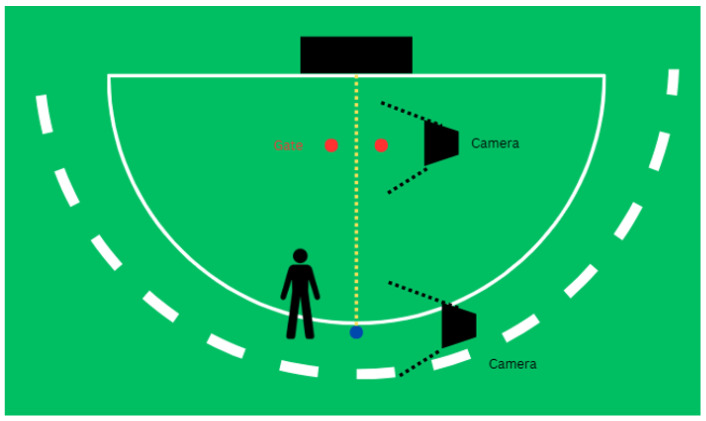
An illustration of the experimental setup. The participant sweeps the ball (blue) from a consistent location just outside of the circle. They are instructed to sweep the ball through the gate (red dots) into the goal (black rectangle). Two cameras are synchronized and collect data as the participant sweeps the ball and as the ball passes through the gate.

Participants were instructed to simulate a penalty corner scenario in which they executed a sweep intended for a deflection between the marked gate. The sweep was executed from a stationary ball positioned 6 inches outside the top of the circle to represent a trapped insert pass, which is a common penalty corner scenario (Figure 1). A 4-foot-wide gate was marked by two colored markers placed even with the stroke mark, which is 7 yards from the goal. To monitor the sweep and the speed of the ball, two GoPro Hero 11 cameras (GoPro, Inc., San Mateo, USA) were placed at the location where the sweep was initiated and where it crossed the marked gate threshold. The cameras recorded data at 120 frames per second (fps) in 4K resolution.

**Figure 2:**
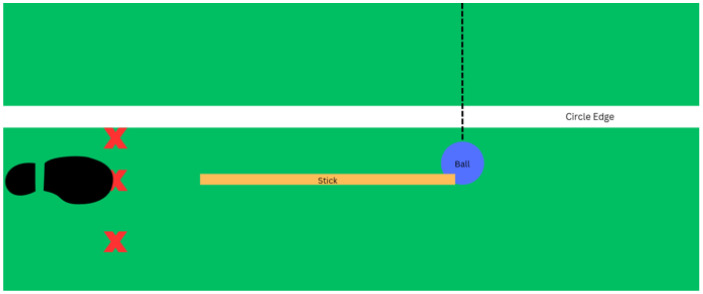
Foot positions relative to the ball. The foot is positioned at the middle red X, which is in line with the ball and is generally regarded as the correct way to complete a sweep. The red X closer to the circle edge is in front of the and the red X farther away from the circle edge is behind the ball. The dashed black line indicates the direction of ball’s motion after impact with the field hockey stick.

Three markers indicating relative foot positions in front of, in line with, and behind the ball were positioned for each participant (Figure 2). When the foot was positioned in front of the ball, it was placed 4 inches closer to the goal than the foot aligned with the ball. When the foot was positioned behind the ball, it was placed 6 inches farther from the goal compared to the in-line foot position. The markers were placed on a plastic mat used by every participant to ensure consistency between trials. The ball was centered to align with the middle of the goal and placed 6 inches beyond the top of the field hockey circle, which is 16 yards from the goal. Each participant indicated their desired horizontal distance from their front foot to the ball, while the alignment of the foot and ball was determined by the research team. For context, field hockey players are frequently coached to place their foot in line with the ball while completing this technical skill. A ball placed behind or in front of the player’s foot is generally believed by coaches and players to result in an unsuccessful sweep. This experimental design choice was intended to investigate the validity of that advice and evaluate how relative foot position influences sweep execution.

**Figure 3:**
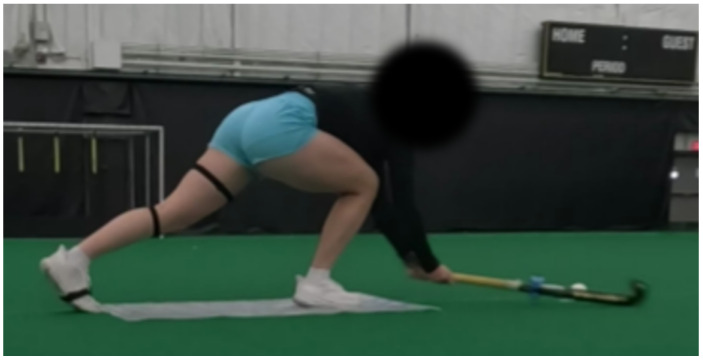
Side view of a participant in the middle of completing a sweep. The stick is about to strike the ball as they fully extend their arms in a lunge position.

Each participant performed the sweep up to 24 times, disregarding any sweeps where the foot was off the intended mark. The sweeps were completed in a randomized order for relative foot position. Not all sweeps were completed successfully (e.g., the ball’s trajectory after being hit was outside of the field of view of both cameras), so there was an unbalanced number of sweeps for each participant in each foot position category. Figure 3 is a still shot of a sweep. As illustrated, players complete the sweep skill in a lunged position. For context, another piece of common coaching advice is to complete the sweep skill as low to the ground as possible. Note that participants completed the sweep while standing on a plastic mat to standardize the relative foot positions as well as to ensure the participants would not slip on the turf.

### 2.3. Inertial Measurement Units

**Figure 4:**
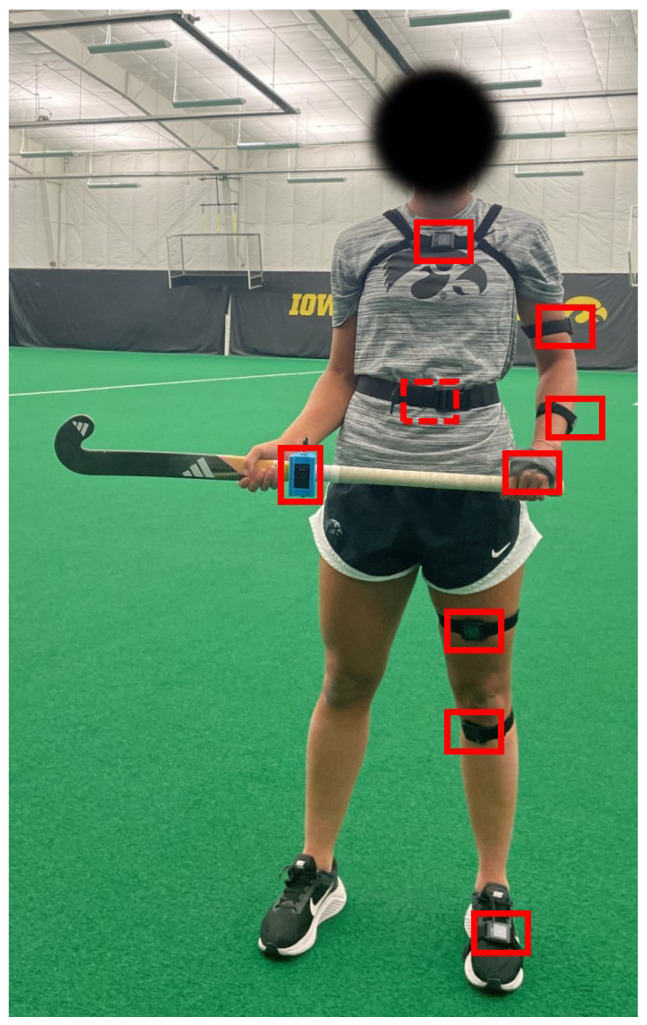
Participant wearing an array of IMUs (red rectangles) attached to major body segments, including the approximate location for the sacrum IMU on posterior side (red dashed rectangle) and the instrumented stick.

**Figure 5:**
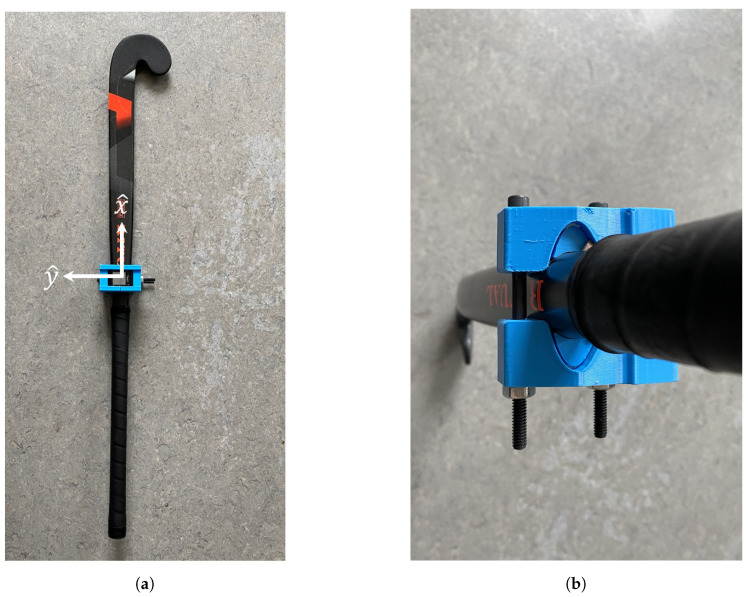
Field hockey stick with a custom 3D printed IMU holder and additional pillows. (**a**) Field hockey stick with IMU holder. (**b**) Longitudinal view of IMU holder.

The inertial measurement units (IMUs) utilized in this study are a commercial design (APDM, Opalv2, Portland, OR, USA) with associated proprietary software. Each IMU includes a triaxial accelerometer (6 g range, 14-bit resolution, 650 μg/$\sqrt{H}$z noise floor) and a triaxial angular rate gyro (2000^°^/s range, 16-bit resolution, 0.03^°^/s/$\sqrt{H}$z noise floor) sampled at 128 Hz. A proprietary Kalman filter provides orientation estimates in quaternion form (see [37] for an example of a Kalman filter implementation). Since all participants executed the sweep from a right-handed stance, IMUs were placed on the left (lead/front) side since the left leg serves as the primary weight-bearing and positioning limb during a right-handed sweep. The participants were fitted with 9 IMUs on the left hand, left forearm, left upper arm, sacrum, sternum, left foot, left shin, left thigh, and stick grip, as illustrated in Figure 4. The stick grip sensor was fastened onto the flat side of the participant’s stick with a custom adjustable and shock-absorbing sensor holder (Figure 5). The sensor holder was 3D printed of thermoplastic polyurethane (TPU) using a Bambu X1 Carbon printer (Bambu Lab, Shenzhen, China). Since participants were encouraged to use a field hockey stick with which they were comfortable, additional TPU pillows were printed to ensure a tight fit with each stick’s geometry (Figure 5b). The quaternion estimates enable each sensor’s body-fixed frame of reference to be related to a world frame.

Once the sensors were placed on the body segments, the sensor-to-body-segment orientation was determined. A static standing calibration was recorded prior to data collection during which participants stood upright with arms at their sides and feet shoulder width apart. It was assumed that the longitudinal axis of each body segment, $\hat{y}$, was aligned with the direction of gravity during this known static standing period. Thus, the accelerometer data were averaged for each IMU to estimate the gravity vector in the sensor frame, which was then normalized to be a unit vector. The resulting unit vector represents an anatomical longitudinal axis resolved in each sensor’s frame, effectively representing a functional alignment procedure [38]. For the stick grip sensor, it was assumed that the sensor-fixed axes were aligned with the relevant dimensions of the stick (Figure 5a).

### 2.4. Data Processing

The two GoPro cameras (GoPro, Inc., San Mateo, CA, USA) were synchronized using a visual cue, in this case tossing an IMU in the air, within the field of view of both cameras at the start of each recording session. The frame corresponding to the moment the IMU left the hand was identified in both video streams and used as a common time reference. The video data were utilized to quantify accuracy, bounciness, and speed of each sweep. Accuracy is defined as a binary variable based on whether the ball passed between the gate markers or not. Bounciness was defined as a binary variable based on whether the ball visibly separated from the ground surface while traveling to the gate or not. Specifically, a sweep was determined to be bouncy if the ball was observed to lose contact with the turf surface. A ball that bounced at any point during the sweep, including within the field of view of the camera, continuously bounced for the entirety of ball travel due to the short ball path and turf surface. Ball speed was calculated by dividing the distance from the ball placement to the gate (27.5 feet) by the ball travel time. Ball travel time was calculated using the time elapsed from the impact with the field hockey stick to the ball crossing the gate. As described above, video data of the participant and of the gate were synchronized prior to calculations to provide an accurate travel time.

The first step in processing the IMU data is automatically parsing the trials by identifying the occurrence of each instance of a sweep. The sweeps are identified by peaks in the linear acceleration magnitude for the IMU attached to the stick since each peak likely corresponds to an impact event between the stick and the ball. The data 1 s before and 1 s following the peak are considered relevant to the sweep.

**Figure 6:**
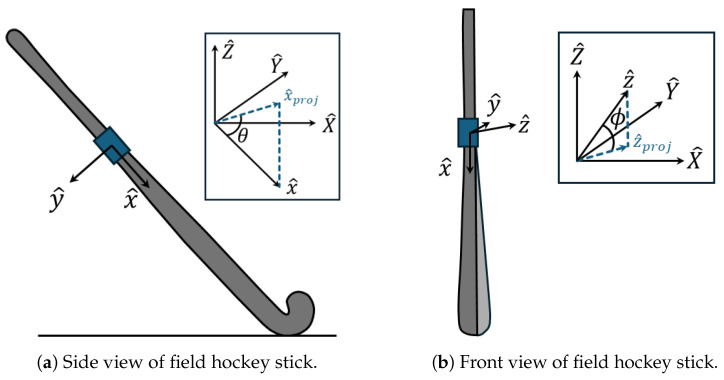
Illustrations for how field hockey stick pitch and roll are calculated. The callouts describe how the body-fixed frame of reference of the stick mounted IMU are related to the world frame.

Next, the pitch and roll for the field hockey stick are derived from the quaternion estimates of the IMU orientation provided by the manufacturer’s software. Pitch represents the angle between the longitudinal axis of the field hockey stick and the horizontal plane. In Figure 6a, the longitudinal axis is represented by a body-fixed axis, $\hat{x}$, which potentially has components in all three directions in the world frame, meaning $\hat{x}={\left[x_{X},x_{Y},x_{Z}\right]}^{T}$. In the callout, $\hat{x}$ is projected onto the horizontal plane of the world frame (i.e., the $\hat{X}-\hat{Y}$ plane) and is denoted ${\hat{x}}_{proj}$. Thus, pitch, denoted by θ, is calculated as follows,

(1) $$\theta =\mathrm{atan}2(x_{Z},||{\hat{x}}_{proj}||)$$

where atan2(·) denotes the four-quadrant inverse tangent function.

Next, roll represents the angle between an axis normal to the flat face of the stick and the horizontal plane. In Figure 6b, the stick is illustrated in a slightly “open” orientation in which the face of the stick is facing upward. The normal axis is represented by another body-fixed axis, $\hat{z}$, which may also have components along all three world frame axes, or $\hat{z}={\left[z_{X},z_{Y},z_{Z}\right]}^{T}$. In the callout, $\hat{z}$ is projected onto the horizontal plane of the world frame, or ${\hat{z}}_{proj}$. Roll, denoted by ϕ, is similarly calculated as follows,

(2) $$\phi =\mathrm{atan}2(z_{Z},||{\hat{z}}_{proj}\left||\right)+90^{{}^{\circ}}$$

where 90^°^ is added for ease of interpretation.

Finally, the participant’s knee and hip flexion–extension angles are calculated also using the quaternion estimates of the IMU orientation. The longitudinal axes of the shank (${\hat{y}}_{sh}$), thigh (${\hat{y}}_{th}$), and sternum (${\hat{y}}_{st}$) are rotated from their respective body-fixed frames of reference into a presumed common world frame. Using the shank as an example, the longitudinal axis for the body segment resolved in the world frame, ${\hat{Y}}_{sh}$, is calculated as follows,

(3) $${\hat{Y}}_{sh}=R_{W/sh}\;{\hat{y}}_{sh}$$

where $R_{W/sh}$ is the rotation matrix representation of the quaternion estimate of IMU orientation. Then, that vector is projected onto the world frame’s horizontal plane (i.e., the $\hat{X}-\hat{Y}$ plane) and normalized to be a unit vector, yielding ${\hat{Y}}_{sh,proj}$. The angles between each body segment’s longitudinal axis and its respective projection are calculated as follows,

(4) $$\begin{array}{c} \alpha =\mathrm{acos}({\hat{Y}}_{sh}^{T}\;{\hat{Y}}_{sh,proj}) \end{array}$$

(5) $$\begin{array}{c} \beta =\mathrm{atan}2(Y_{th,Z},\;||{\hat{Y}}_{th,proj}||) \end{array}$$

(6) $$\begin{array}{c} \psi =\mathrm{acos}({\hat{Y}}_{st}^{T}\;{\hat{Y}}_{st,proj}) \end{array}$$

where acos(·) is the inverse cosine function that returns values between $\left[0^{{}^{\circ}},180^{{}^{\circ}}\right]$ and $Y_{th,Z}$ is the *Z*-component of the thigh’s longitudinal axis resolved in the world frame. The knee and hip flexion–extension angles are calculated as follows,

(7) $$\begin{array}{c} \mathrm{knee}=\alpha +\beta \end{array}$$

(8) $$\begin{array}{c} \mathrm{hip}=\psi +\beta \end{array}$$

where the sign associated with the thigh’s orientation, β, is conserved via the atan2(·) function. If β is greater (less) than $0^{{}^{\circ}}$, the thigh is above (below) the horizontal plane.

### 2.5. Statistical Analysis

As stated above, this exploratory study hypothesized that relative foot position, stick orientation, and joint angles at impact with the ball will influence the performance of the sweep skill. For the purposes of this study, a superior sweep is defined as one that is accurate, flat, and fast. The first hypothesis (H1) is that a sweep performed with the relative foot position (RFP) in line with the ball will lead to a superior sweep compared to sweeping with the foot in front or behind the ball. The second hypothesis (H2) is that a stick that is closed and low to the ground at the moment of impact will lead to a superior sweep. A closed stick orientation is one in which the roll angle is less than $90^{{}^{\circ}}$ and a low stick orientation is one in which the pitch angle is as close to $0^{{}^{\circ}}$ as possible. The third and final hypothesis (H3) is that a low stance at the moment of impact will lead to a superior sweep. A low stance is defined as knee and hip angles that are as small as possible, indicating that the joints are substantially flexed; for reference, standing upright would result in joint angles close to $180^{{}^{\circ}}$.

To test these hypotheses, mixed-effects regression analyses are conducted for each of the three dependent variables (accuracy, bounciness, and ball speed) as follows,

(9) Outcome∼1+RFP+θ+ϕ+knee+hip+(1|participant)

where the model includes a potentially important intercept (‘1’) associated with the population averages when the relative foot position is in line with the ball. The random effects variable (1|participant) accounts for differences between participants. Accuracy and bounciness (binary outcomes) were analyzed using logistic mixed-effects regression, while ball speed (continuous) was analyzed using linear mixed-effects regression. Interaction terms were considered but not included as they were not specified in the study hypotheses and would increase model complexity without theoretical justification. The model included one categorical predictor, RFP (three levels with “In Line” as the reference), and four continuous predictors (θ, ϕ, knee angle, and hip angle). Continuous variables were mean-centered to facilitate interpretation of the intercept. For logistic regressions, coefficients are reported as log-odds, with corresponding odds ratios and 95% confidence intervals provided. Statistical significance was evaluated at α=0.05.

To further aid interpretation of the mixed-effects results, an additional within–between decomposition analysis was also conducted in which predictors were separated into within-participant deviation scores and between-participant mean differences. This analysis was performed to distinguish trial-level effects from stable individual differences that may otherwise be conflated in the mixed-effects models. Results are presented in Appendix A as a robustness and interpretability check and do not replace the primary aforementioned analysis. All data processing and statistical analyses were performed in MATLAB (R2024a, MathWorks, Natick, MA, USA).

## 3. Results

All analyses were conducted at the level of individual sweeps, with each sweep representing a single interaction between the field hockey stick and ball, forming one observation in the results to follow. In total, the participants completed 171 viable sweeps. Twelve sweeps were discarded due to either the participant’s foot being placed off the intended mark or to a sweep with an extremely inaccurate trajectory that was unable to be seen in the frame of the gate camera. An additional 9 sweeps (∼5% of all sweeps) were excluded due to physically impossible values resulting from transient IMU movement at impact.

### 3.1. Overall Sweep Performance

Table 1 includes the overall performance for the sweep skills for each foot position relative to the ball placement. For accuracy, a value of 0 indicates the sweeps were inaccurate and a value of 1 indicates the sweeps were accurate. For bounciness, a value of 0 indicates the sweeps were flat and a value of 1 indicates the sweeps were bouncy. For these binary variables, a higher standard deviation means higher mix in the sample, with the maximum possible value being 0.5.

### 3.2. Field Hockey Stick Pitch and Roll

**Figure 7:**
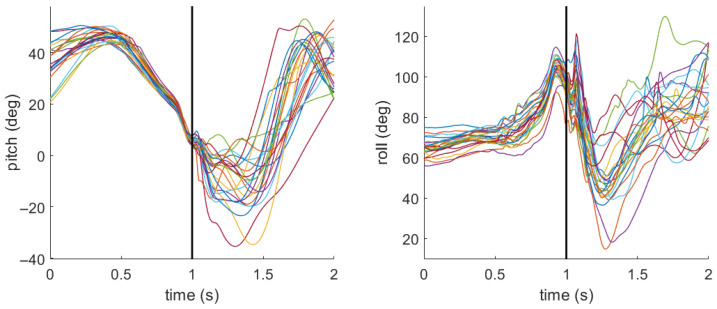
Pitch and roll stick profiles for a participant with overall low (18%) sweep accuracy. The black vertical line denotes the moment of impact between the stick and ball. Each colored line represents an individual sweep.

**Figure 8:**
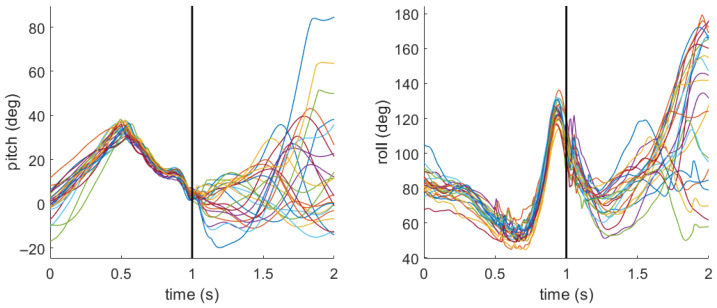
Pitch and roll stick profiles for a participant with overall high (95%) sweep accuracy. The black vertical line denotes the moment of impact between the stick and ball. Each colored line represents an individual sweep.

Figure 7 and Figure 8 include the pitch and roll profiles for the field hockey stick during all sweeps completed by two different participants. For the sake of comparison, the profiles were chosen for the participants with the lowest and highest overall sweep accuracy. For the participant with low sweep accuracy (Figure 7), the mean (standard deviation) for pitch was 5.2 deg (1.4 deg) and for roll it was 96.3 deg (6.3 deg) at the moment of impact. For the participant with high sweep accuracy (Figure 8), the mean (standard deviation) for pitch was 4.0 deg (1.6 deg) and for roll it was 107.6 deg (6.5 deg).

Table 2 includes the stick pitch and roll at the impact with the ball for each foot position relative to the location of the ball. For pitch, a value closer to 0 indicates that the stick is closer to horizontal. For roll, a value closer to 90 indicates that the flat face of the stick is normal to the impact direction with the ball. Values greater than 90 indicate the flat face is open, or facing upward, whereas values less than 90 indicate the flat face is closed, or facing downward. Interpretation of stick orientation at impact should be made with caution, as a small number of trials were excluded due to impact related IMU artifacts, which suggests that measurements at this instant may be sensitive to transient sensor disturbances.

### 3.3. Joint Angles

**Figure 9:**
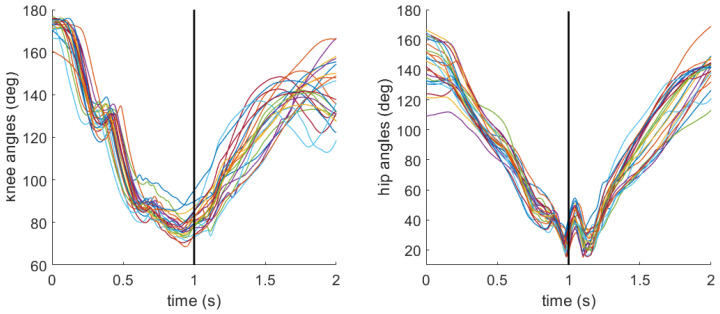
Knee and hip angle profiles for a participant with overall low (18%) sweep accuracy. The black vertical line denotes the moment of impact between the stick and ball. Each colored line represents an individual sweep.

**Figure 10:**
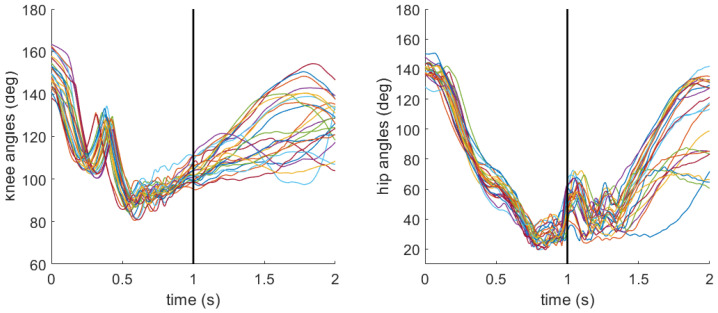
Knee and hip angle profiles for a participant with overall high (95%) sweep accuracy. The black vertical line denotes the moment of impact between the stick and ball. Each colored line represents an individual sweep.

As a reminder, joint angles at the knee and hip were calculated to characterize the participant’s stance at impact. Angles were defined such that smaller values correspond to greater joint flexion, representing a lower stance, while larger values indicate a more extended (i.e., upright) posture. Figure 9 and Figure 10 include the knee and hip angle profiles during all sweeps completed by the same two aforementioned participants. For the participant with low sweep accuracy, the mean (standard deviation) for knee angle was 81.1 deg (5.2 deg), and for hip angle was 29.6 deg (6.5 deg) at the moment of impact. For the participant with high sweep accuracy, the mean (standard deviation) for knee angle was 103.8 deg (4.3 deg), and for hip angle was 48.8 deg (8.5 deg).

Table 3 includes the knee and hip angles at the impact with the ball for each foot position relative to the ball location, where smaller angles (i.e., greater flexion) correspond to a lower stance.

### 3.4. Statistical Analysis Results

Appendix A presents the results from additional within–between decomposition analyses that complement the mixed-effects analyses presented here. Table 4 reports the results from the logistic mixed-effects regression analysis where accuracy is the binary dependent variable (0 indicates inaccurate and 1 indicates accurate). As a reminder, the relative foot position (RFP) “In Line” with the ball served as the reference category, which means that the intercept corresponds to the predicted outcome for this condition when all continuous predictors are at their mean values.

Table 5 reports the results from the logistic mixed-effects regression analysis where bounciness is the binary dependent variable (0 indicates flat and 1 indicates bouncy).

Table 6 reports the results from the linear mixed-effects regression analysis where ball speed is the continuous dependent variable.

## 4. Discussion

This study’s first hypothesis assumed that sweeps completed when the foot was in line with the ball would result in superior performance (more accurate, more flat, and faster) than either of the other two foot positions (H1). Starting with overall performance (Table 1), about 66% of the sweeps that the participants completed were accurate. Achieving a flat sweep was more challenging since about 62% of sweeps were classified as bouncy. Finally, average ball speed was about 48 ft/s, which means the ball travels from the edge of the field hockey circle to the goal in about 1 s. Across all three metrics, completing a sweep with the foot position in front of the ball descriptively exhibited worse performance on average, offering initial support for H1.

From the results presented in Table 4, sweeping when the foot is in front of the ball trended towards reduced odds of accuracy relative to the in-line reference condition (β = −0.92, *p* = 0.05). The corresponding odds ratio was 0.40, indicating that placing the foot in front of the ball may reduce the odds of an accurate sweep by approximately 60%, though this effect sits at the boundary of conventional statistical significance. By contrast, sweeping with the foot behind the ball did not differ significantly from the reference condition (β = −0.37, *p* = 0.43). For this model, the intercept was positive and significant (β = 1.30, *p* = 0.01), which means that when the foot is positioned in line with the ball and the continuous predictors are at their average values, the predicted probability of an accurate sweep is approximately 79%. Next, Table 5 shows that foot position did not significantly predict bounciness. Sweeping with the foot in front of the ball trended towards lower odds of bounciness relative to when the foot was in line with the ball (β = −0.77, *p* = 0.10, OR = 0.46), whereas placing the foot behind the ball did not differ from the reference (β = 0.37, *p* = 0.40, OR = 1.45). When the foot is positioned in line with the ball and the continuous predictors are at their reference values, the model-predicted probability of the ball being bouncy is approximately 37% (β = −0.47, OR = 0.63, *p* = 0.25). With respect to Table 6, sweeping with the foot placed in front of the ball was associated with a significant reduction in ball speed (∼2.8 ft/s) compared with the reference condition (β = −2.8, *p* < 0.01), holding all other predictors constant. Sweeping with the foot behind the ball did not differ significantly from the reference (β = −0.7, *p* = 0.45). Collectively, these results suggest that foot position relative to ball placement influences overall sweep performance, primarily through reduced ball speed with a trend for reduced accuracy. Additionally, the findings support only that placing the foot in front of the ball leads to inferior performance whereas placing the foot behind the ball leads to nominally similar performance.

The second hypothesis (H2) stated that a closed and low stick orientation at impact would result in superior performance. Reflecting on the results displayed in Figure 7 and Figure 8, the participant with overall high sweep accuracy goes from a relatively closed stick orientation (∼50^°^) about 0.4 s before impact to a relatively open stick orientation (∼130^°^) just before impact. The participant with overall low sweep accuracy exhibits similar behavior, but the angles are not as extreme. Roll angle showed no evidence of a significant effect on accuracy (β = 0.01, *p* = 0.96, OR ≈ 1). However, roll angle was the only statistically significant predictor of bounciness (β = 0.12, *p* < 0.001, OR = 1.13), indicating that each $1^{{}^{\circ}}$ increase in roll angle at impact was associated with approximately a 13% increase in the odds of a bouncy sweep. This finding was corroborated by the within–between decomposition (Table A2), where the within-participant roll effect remained significant (β = 0.11, *p* < 0.01), indicating that this finding is a trial-level effect rather than merely a stable individual difference. Roll angle was also a significant positive predictor of ball speed (β = 0.2, *p* < 0.01), suggesting that a more open stick face at impact is associated with modestly faster sweeps. Referring to Table 2, the average roll angle when the foot was in line with the ball was $100.8^{{}^{\circ}}$, which indicates that the stick face is on average slightly open. However, in observing the roll times series profiles in Figure 7 and Figure 8, the stick is rapidly changing from an open face to a closed face, with the impact happening during that motion. This behavior is likely attributable to the participant’s wrists rolling over through the impact phase. It may be the case that the timing of that wrist rolling motion is important to achieve a flat, faster sweep. With respect to pitch, the primary model found no significant association with accuracy, bounciness, or ball speed (Table 4, Table 5 and Table 6). Both participants have very consistent pitch profiles leading up to the impact, though they have very different follow through strategies (Figure 7 and Figure 8). However, the between-participant decomposition revealed that players who tend to impact the ball at higher pitch angles on average hit the ball more slowly (β = –2.0, *p* < 0.01; Table A3). Overall, roll angle consistently shaped bounciness and ball speed outcomes, although these results should be interpreted with some caution given that stick orientation was estimated at impact where transient IMU disturbances can occur. Pitch angle effects on ball speed appear to operate primarily as a between-player attribute rather than a within-player, trial-to-trial phenomenon. Together, these results provide partial support for H2.

The final hypothesis (H3) posited that a lower stance at impact would result in superior sweep performance. From the time series data, the participant with overall high sweep accuracy appears to exhibit decreases in knee and hip angles leading up to the impact (Figure 10) compared with the participant with overall low sweep accuracy (Figure 9). However, neither knee nor hip angle reached statistical significance for any of the three outcomes (Table 4, Table 5 and Table 6). However, the within–between decomposition provides important nuance. At the between-participant level, larger knee angles (less flexion) were associated with increased odds of accuracy (OR = 1.14, *p* = 0.03), and smaller hip angles (greater flexion) were associated with reduced odds of accuracy (OR = 0.85, *p* < 0.01; Table 4). No significant between- or within-participant effects of knee or hip angle were found for bounciness or ball speed. Together, these results offer only partial support for H3. Lower limb posture does appear to be related to accuracy, but the relationship is driven by stable between-participant differences in habitual stance rather than trial-level adjustments and does not extend meaningfully to bounciness or ball speed.

Previous sweep literature considered the angles and moments of the players’ joints during the sweep based on ball positioning [36]. The results of that study revealed that placing the ball in line with the foot resulted in the lowest moments and angles in the knee joint as opposed to placing it in front of or behind the foot. The current findings indicate that placing the ball in line with the foot leads to favorable accuracy and ball speed, while placing the ball behind the foot does not lead to significantly different results from the in-line position. The present findings suggest that instructing players to sweep the ball in line with their front foot may be associated with a relatively lower risk of injury while maintaining favorable sweep quality based on prior biomechanical evidence [36].

While common field hockey coaching points instruct players to bend their knees to achieve a better sweep, the present results suggest that the relationship between a lower stance and sweep performance is more nuanced than a simple instruction to “get lower”. The between-participant decomposition indicates that players who exhibit larger knee angles and smaller hip angles tend to be more accurate, yet these are stable stylistic tendencies rather than adjustments players can make on a trial-by-trial basis. At the stick level, roll angle emerged as the most actionable trial-level predictor. A more open face at impact consistently increased both bounciness and ball speed, which aligns with a common coaching cue to avoid opening the stick. The time-series profiles further suggest that accurate sweeps are characterized by a closed stick leading into contact, followed by a rapid opening and closing of roll angle at impact, which is likely driven by a wrist snap through the ball. This dynamic wrist action, rather than a statically closed face, appears to be characteristic of effective sweeps. Coaching instruction might therefore benefit from emphasizing controlled wrist rotation through contact rather than a fixed stick orientation, while also recognizing that slightly more open roll angles can modestly increase ball speed at the cost of an increased likelihood of a bouncy sweep. The optimal stick orientation at impact is therefore likely to vary depending on the intended outcome and game context.

### Current Limitations and Future Work

The current study has several limitations. The sample included only eight experienced players, limiting the precision of between-participant estimates and reducing generalizability. Sweeps were performed in a controlled setting that did not fully replicate gameplay conditions, and although IMUs provided rich kinematic data, their accuracy depends on calibration and filtering assumptions that may introduce small errors during rapid stick motion. In particular, the estimation of stick orientation at impact may be affected by transient IMU motion during ball contact. Although trials with clearly implausible values were excluded, smaller unobservable artifacts may still influence pitch and roll estimates, warranting cautious interpretation of these variables. This sensitivity highlights the need for future work to further validate IMU-derived stick orientation at impact. The statistical models were intentionally additive to avoid overfitting, which may omit higher order interactions among variables. For example, it is possible that relative foot placement and participant height drive changes in stick orientation and joint angle behaviors. Finally, only a subset of potential biomechanical factors was measured. Additional variables such as trunk rotation, limb coordination, or the third rotational component of the stick may also influence sweep performance.

Future work should consider recruiting a larger, more diverse cohort. In parallel, moving from a controlled setup with a stationary ball to more relevant gameplay conditions would better capture the kinematics and outcomes that arise in competition. Intervention studies that alter coaching cues (e.g., emphasizing knee- versus hip-dominant lowering strategies) and track subsequent performance changes would help establish causal links between technique adjustments and sweep outcomes, thereby translating these biomechanical insights into practical training recommendations. Additionally, future studies should more fully exploit the time-series richness of IMU signals by examining pre-impact kinematic trajectories using feature-based or functional data analysis approaches, which may reveal dynamic patterns not captured by discrete measures derived at the moment of impact between the field hockey stick and ball. Finally, future work should also investigate how the biomechanical behaviors studied here relate to known mechanisms of lower body and lumbar loading in field hockey, thereby linking sweep technique with injury risk reduction and informing evidence-based prevention efforts.

## 5. Conclusions

In summary, this exploratory study provides initial insight into how relative foot placement, stick orientation, and lower body kinematics at impact may meaningfully influence field hockey sweep performance. Foot position influenced performance selectively. Placing the foot in front of the ball was associated with a trend toward reduced accuracy and a significant reduction in ball speed relative to the in-line reference condition, whereas placing the foot behind the ball did not differ meaningfully from in line across any outcome. Stick roll angle at impact emerged as the most consistent observed trial-level predictor with a more open face associated with increased bounciness and modestly faster ball speed, although estimates at impact may be sensitive to transient IMU artifacts and should be interpreted cautiously. Stick pitch and lower limb joint angles did not act as significant within-participant predictors. However, within–between analyses indicated that larger knee angles and smaller hip angles were associated with improved accuracy, while smaller pitch angles were associated with faster ball speed. Together, these findings suggest that some aspects of sweep performance, particularly stick roll, are more amenable to trial-level technique adjustments, whereas others reflect stable individual movement tendencies that may be better addressed through longer-term coaching or motor learning interventions. Because lower body posture and trunk orientation are also linked to loading patterns implicated in overuse injuries, these results provide a starting point for future work examining how technique may enhance performance while also informing injury prevention efforts in field hockey.

## Figures and Tables

**Table 1:** Mean and standard deviation (SD) of sweep performance metrics by relative foot position (RFP). Accuracy and bounciness are reported as proportions. Higher accuracy indicates more trials passing through the gate. Higher bounciness indicates more trials with ball-ground separation.

RFP	Accuracy	Bounciness	Speed [ft/s]
In Front	0.54±0.50	0.26±0.44	46.0±8.5
In Line	0.71±0.46	0.42±0.50	49.6±6.9
Behind	0.71±0.46	0.47±0.50	48.7±6.8

**Table 2:** Mean and standard deviation (SD) of pitch and roll angles at impact across participants by relative foot position (RFP).

RFP	Pitch [deg]	Roll [deg]
In Front	6.1±3.1	100.1±9.3
In Line	5.4±2.6	100.8±7.7
Behind	6.3±2.2	102.8±7.0

**Table 3:** Mean and standard deviation (SD) of knee and hip angles at impact across participants by relative foot position (RFP).

RFP	Knee Angle [deg]	Hip Angle [deg]
In Front	92.9±12.5	29.2±12.0
In Line	95.8±13.0	34.8±14.0
Behind	97.8±13.8	34.0±15.6

**Table 4:** Logistic mixed-effects regression model estimating the probability of an accurate sweep. Log-odds (β) are raw regression coefficients. Odds ratios (ORs) are exponentiated coefficients. CI:LB and CI:UB indicate the lower and upper bounds of the 95% confidence interval for the odds ratio. Standard errors (SEs) and *p*-values are also included. For RFP, coefficients represent contrasts relative to the reference category (In Line).

Term	*β*	OR	CI:LB	CI:UB	SE	*p*-Value
***Fixed Effects***						
**Intercept**	1.30	3.68	1.35	10.02	0.51	0.01 *
**RFP**						
In Front	−0.92	0.40	0.16	1.00	0.47	0.05 **
Behind	−0.37	0.69	0.27	1.75	0.47	0.43
**Stick Orientation**						
Pitch	−0.03	0.97	0.78	1.22	0.11	0.81
Roll	0.01	1.00	0.95	1.05	0.03	0.96
**Joint Angles**						
Knee	0.06	1.06	0.99	1.14	0.04	0.10
Hip	−0.04	0.96	0.90	1.02	0.03	0.17
***Random Effects***						
Participant (intercept SD)	1.06 (variance = 1.12)					

The statistical significant is reported for the following levels: * *p* < 0.05, ** *p* < 0.10.

**Table 5:** Logistic mixed-effects regression model estimating the probability of a bouncy sweep. Log-odds (β) are raw regression coefficients. Odds ratios (ORs) are exponentiated coefficients. CI:LB and CI:UB indicate the lower and upper bounds of the 95% confidence interval for the odds ratio. Standard errors (SEs) and *p*-values are also included. For RFP, coefficients represent contrasts relative to the reference category (In Line).

Term	*β*	OR	CI:LB	CI:UB	SE	*p*-Value
***Fixed Effects***						
**Intercept**	−0.47	0.63	0.28	1.39	0.40	0.25
**RFP**						
In Front	−0.77	0.46	0.18	1.16	0.47	0.10
Behind	0.37	1.45	0.61	3.45	0.44	0.40
**Stick Orientation**						
Pitch	0.02	1.02	0.82	1.27	0.11	0.88
Roll	0.12	1.13	1.06	1.20	0.03	<0.001 ^‡^
**Joint Angles**						
Knee	−0.05	0.95	0.89	1.02	0.03	0.15
Hip	0.02	1.02	0.97	1.08	0.03	0.40
***Random Effects***						
Participant (intercept SD)	0.74 (variance = 0.54)					

The statistical significant is reported for the following levels: ^‡^ *p* < 0.001.

**Table 6:** Linear mixed-effects regression model estimating ball speed. Coefficients are shown with 95% lower and upper confidence interval bounds (CI:LB and CI:UB, respectively), standard errors (SEs), and corresponding *p*-values.

Term	*β*	CL:LB	CL:UB	SE	*p*-Value
***Fixed Effects***					
**Intercept**	49.5	46.0	52.9	1.8	<0.001 ^‡^
**RFP**					
In Front	−2.8	−4.6	−0.9	0.9	<0.01 ^†^
Behind	−0.7	−2.5	1.1	0.9	0.45
**Stick Orientation**					
Pitch	−0.3	−0.8	0.2	0.3	0.31
Roll	0.2	0.1	0.3	0.1	<0.01 ^†^
**Joint Angles**					
Knee	−0.1	−0.2	0.1	0.1	0.70
Hip	0.1	−0.1	0.2	0.1	0.81
***Random Effects***					
Participant	4.63	2.68	7.97	-	-

The statistical significant is reported for the following levels: ^†^ *p* < 0.01, ^‡^ *p* < 0.001.

## Data Availability

The de-identified datasets may be available upon reasonable request.

## Appendix A

Decomposed mixed-effects regression models were used to examine associations between foot position and kinematic variables (i.e., pitch, roll, knee angle, and hip angle) and three outcomes: accuracy, bounciness, and ball speed. The binary outcomes (accuracy and bounciness) were modeled using logistic mixed-effects regression, while ball speed was modeled using a linear mixed-effects regression. For all models, fixed effects included foot position and decomposed kinematic predictors, each represented as a within-participant deviation term and a between-participant mean term. This decomposition enabled separate estimation of trial-level (within-person) effects and stable individual differences (between-person effects) that may otherwise be conflated in standard mixed-effects models. A random intercept for participant was included to account for repeated observations. Model coefficients, standard errors, and p-values were extracted from the fitted models. For logistic models, odds ratios and 95% confidence intervals were obtained by exponentiating the coefficient estimates and their confidence bounds. As highlighted above, these analyses are presented as a robustness and interpretability check and do not replace the primary analyses reported in the main text.

**Table A1:** Decomposition logistic mixed-effects regression model estimating the probability of an accurate sweep. Log-odds (β) are raw regression coefficients. Odds ratios (ORs) are exponentiated coefficients. CI:LB and CI:UB indicate the lower and upper bounds of the 95% confidence interval for the odds ratio. Standard errors (SEs) and *p*-values are also included. For RFP, coefficients represent contrasts relative to the reference category (In Line).

Term	*β*	OR	CI:LB	CI:UB	SE	*p*-Value
***Fixed Effects***						
**Intercept**	1.26	3.53	1.48	8.41	0.44	<0.01 ^†^
**RFP**						
In Front	−0.92	0.40	0.16	1.00	0.47	0.05 **
Behind	−0.39	0.68	0.27	1.74	0.48	0.42
**Within Participant Effects**						
Pitch	−0.01	1.00	0.77	1.30	0.13	0.99
Roll	0.01	1.00	0.95	1.06	0.03	0.88
Knee	0.06	1.07	0.97	1.18	0.05	0.20
Hip	−0.02	0.98	0.91	1.04	0.03	0.47
**Between Participant Effects**						
Pitch	0.27	0.76	0.49	1.18	0.22	0.22
Roll	0.13	1.14	0.90	1.45	0.12	0.27
Knee	0.13	1.14	1.01	1.28	0.06	0.03 *
Hip	−0.17	0.85	0.75	0.96	0.06	<0.01 ^†^
***Random Effects***						
Participant (intercept SD)	0.77 (variance = 0.59)					

The statistical significant is reported for the following levels: ** *p* < 0.10, * *p* < 0.05, ^†^ *p* < 0.01.

**Table A2:** Decomposition logistic mixed-effects regression model estimating the probability of a bouncy sweep. Log-odds (β) are raw regression coefficients. Odds ratios (ORs) are exponentiated coefficients. CI:LB and CI:UB indicate the lower and upper bounds of the 95% confidence interval for the odds ratio. Standard errors (SEs) and *p*-values are also included. For Foot Position, coefficients represent contrasts relative to the reference category (In Line).

Term	*β*	OR	CI:LB	CI:UB	SE	*p*-Value
***Fixed Effects***						
**Intercept**	−0.45	0.64	0.29	1.39	0.39	0.26
**Foot Position**						
In Front	−0.76	0.47	0.18	1.19	0.47	0.11
Behind	0.37	1.45	0.60	3.49	0.44	0.41
**Within Participant Effects**						
Pitch	0.10	1.11	0.83	1.48	0.15	0.48
Roll	0.11	1.11	1.04	1.19	0.03	<0.01 ^†^
Knee	−0.05	0.95	0.86	1.06	0.05	0.39
Hip	0.01	1.01	0.95	1.08	0.03	0.71
**Between Participant Effects**						
Pitch	−0.07	0.93	0.62	1.40	0.21	0.74
Roll	0.11	1.11	0.89	1.39	0.11	0.34
Knee	−0.05	0.95	0.86	1.06	0.05	0.37
Hip	0.03	1.03	0.92	1.16	0.06	0.58
***Random Effects***						
Participant (intercept SD)	0.70 (variance = 0.49)					

The statistical significant is reported for the following levels: ^†^ *p* < 0.01.

**Table A3:** Decomposed linear mixed-effects regression model estimating ball speed. Coefficients (β) are shown with 95% lower and upper confidence interval bounds (CI:LB and CI:UB, respectively), standard errors (SEs), and corresponding *p*-values. For Foot Position, coefficients represent contrasts relative to the reference category (In Line).

Term	*β*	CI:LB	CI:UB	SE	*p*-Value
***Fixed Effects***					
**Intercept**	49.5	47.0	51.9	1.2	<0.001 ^‡^
**Foot Position**					
In Front	−2.7	−4.5	−0.8	0.9	<0.01 ^†^
Behind	−0.7	−2.5	1.1	0.9	0.47
**Within Participant Effects**					
Pitch	−0.1	−0.7	0.4	0.3	0.65
Roll	0.2	0.1	0.3	0.1	0.01 *
Knee	−0.01	−0.2	0.2	0.1	0.97
Hip	−0.01	−0.1	0.1	0.1	0.94
**Between Participant Effects**					
Pitch	−2.0	−3.5	−0.5	0.8	<0.01 ^†^
Roll	−0.4	−1.2	0.4	0.4	0.37
Knee	0.01	−0.4	0.4	0.2	0.96
Hip	0.1	−0.3	0.5	0.2	0.68
***Random Effects***					
Participant	3.0	1.8	5.2	-	-

The statistical significant is reported for the following levels: * *p* < 0.05, ^†^ *p* < 0.01, ^‡^ *p* < 0.001.

## References

[1] Malm C., Jakobsson J., Isaksson A. (2019). Physical activity and sports—real health benefits: A review with insight into the public health of Sweden. Sports.

[2] Kjønniksen L., Anderssen N., Wold B. (2009). Organized youth sport as a predictor of physical activity in adulthood. Scand. J. Med. Sci. Sports.

[3] Eime R.M., Young J.A., Harvey J.T., Charity M.J., Payne W.R. (2013). A systematic review of the psychological and social benefits of participation in sport for children and adolescents: Informing development of a conceptual model of health through sport. Int. J. Behav. Nutr. Phys. Act..

[4] Holt N.L., Kingsley B.C., Tink L.N., Scherer J. (2011). Benefits and challenges associated with sport participation by children and parents from low-income families. Psychol. Sport Exerc..

[5] Rice S.M., Purcell R., De Silva S., Mawren D., McGorry P.D., Parker A.G. (2016). The mental health of elite athletes: A narrative systematic review. Sports Med..

[6] Fortier K., Parent S., Lessard G. (2020). Child maltreatment in sport: Smashing the wall of silence: A narrative review of physical, sexual, psychological abuses and neglect. Br. J. Sports Med..

[7] Johnson N., Hanna K., Novak J., Giardino A.P. (2020). US center for SafeSport: Preventing abuse in sports. Women Sport Phys. Act. J..

[8] Joy E., Kussman A., Nattiv A. (2016). 2016 update on eating disorders in athletes: A comprehensive narrative review with a focus on clinical assessment and management. Br. J. Sports Med..

[9] Soligard T., Schwellnus M., Alonso J.M., Bahr R., Clarsen B., Dijkstra H.P., Gabbett T., Gleeson M., Hägglund M., Hutchinson M.R. (2016). How much is too much? (Part 1) International Olympic Committee consensus statement on load in sport and risk of injury. Br. J. Sports Med..

[10] Schwellnus M., Soligard T., Alonso J.M., Bahr R., Clarsen B., Dijkstra H.P., Gabbett T.J., Gleeson M., Hägglund M., Hutchinson M.R. (2016). How much is too much? (Part 2) International Olympic Committee consensus statement on load in sport and risk of illness. Br. J. Sports Med..

[11] Seçkin A.Ç., Ateş B., Seçkin M. (2023). Review on Wearable Technology in sports: Concepts, Challenges and opportunities. Appl. Sci..

[12] Gabbett T.J. (2016). The training—Injury prevention paradox: Should athletes be training smarter and harder?. Br. J. Sports Med..

[13] Seshadri D.R., Li R.T., Voos J.E., Rowbottom J.R., Alfes C.M., Zorman C.A., Drummond C.K. (2019). Wearable sensors for monitoring the internal and external workload of the athlete. npj Digit. Med..

[14] Wilkerson G.B., Gupta A., Allen J.R., Keith C.M., Colston M.A. (2016). Utilization of practice session average inertial load to quantify college football injury risk. J. Strength Cond. Res..

[15] Preatoni E., Bergamini E., Fantozzi S., Giraud L.I., Orejel Bustos A.S., Vannozzi G., Camomilla V. (2022). The use of wearable sensors for preventing, assessing, and informing recovery from sport-related musculoskeletal injuries: A systematic scoping review. Sensors.

[16] Adesida Y., Papi E., McGregor A.H. (2019). Exploring the role of wearable technology in sport kinematics and kinetics: A systematic review. Sensors.

[17] Li R.T., Salata M.J., Rambhia S., Sheehan J., Voos J.E. (2020). Does overexertion correlate with increased injury? The relationship between player workload and soft tissue injury in professional American football players using wearable technology. Sports Health.

[18] Alzahrani A., Ullah A. (2024). Advanced biomechanical analytics: Wearable technologies for precision health monitoring in sports performance. Digit. Health.

[19] Murtaugh K. (2001). Injury patterns among female field hockey players. Med. Sci. Sports Exerc..

[20] Powell J.W., Barber-Foss K.D. (1999). Injury patterns in selected high school sports: A review of the 1995–1997 seasons. J. Athl. Train..

[21] Murtaugh K. (2009). Field hockey injuries. Curr. Sports Med. Rep..

[22] van Hilst J., Hilgersom N.F., Kuilman M.C., Kuijer P.P.F., Frings-Dresen M.H. (2015). Low back pain in young elite field hockey players, football players and speed skaters: Prevalence and risk factors. J. Back. Musculoskelet. Rehabil..

[23] Barboza S.D., Joseph C., Nauta J., Van Mechelen W., Verhagen E. (2018). Injuries in field hockey players: A systematic review. Sports Med..

[24] Cornelissen M., Kemler E., Verhagen E., Gouttebarge V. (2020). A systematic review of injuries in recreational field hockey: From injury problem to prevention. J. Sports Sci..

[25] Wilmes E., de Ruiter C.J., Beers L.G., de Koning L., Brink M.S., Savelsbergh G.J. (2023). New training load metrics in field hockey using inertial measurement units. Eur. J. Sport Sci..

[26] Wilmes E., de Ruiter C.J., van Leeuwen R.R., Banning L.F., van der Laan D., Savelsbergh G.J. (2024). Different aspects of physical load in small-sided field hockey games. J. Strength Cond. Res..

[27] Latino F., Tafuri F. (2024). Wearable sensors and the evaluation of physiological performance in elite field hockey players. Sports.

[28] Sharma M., Singh A., Shenoy S. (2024). Biomechanical comparison of hip and ankle kinematics among national and university-level field hockey players: An observational study. MGM J. Med. Sci..

[29] Willmott A.P., Dapena J. (2012). The planarity of the stickface motion in the field hockey hit. J. Sports Sci..

[30] Brétigny P., Leroy D., Button C., Chollet D., Seifert L. (2011). Coordination profiles of the expert field hockey drive according to field roles. Sports Biomech..

[31] Brétigny P., Seifert L., Leroy D., Chollet D. (2008). Upper-limb kinematics and coordination of short grip and classic drives in field hockey. J. Appl. Biomech..

[32] López de Subijana C., Juárez D., Mallo J., Navarro E. (2010). Biomechanical analysis of the penalty-corner drag-flick of elite male and female hockey players. Sports Biomech..

[33] López de Subijana C., Gómez M., Martin-Casado L., Navarro E. (2012). Training-induced changes in drag-flick technique in female field hockey players. Biol. Sport.

[34] Ng L., Sherry D., Loh W.B., Sjurseth A.M., Iyengar S., Wild C., Rosalie S. (2016). The prevalence and severity of injuries in field hockey drag flickers: A retrospective cross-sectional study. J. Sports Sci..

[35] Ladru B.J., Beddows T., Langhout R., Gijssel M., Tak I. (2025). What biomechanical parameters are related to drag-flick performance in field hockey? A systematic review. Sports Biomech..

[36] Aikman J.N., Arnold G.P., Nasir S., Wang W.W., Abboud R. (2019). Effect of ball position on the risk of injury to the lower limb joints during the hockey sweep pass in women. BMJ Open Sport Exerc. Med..

[37] Vitali R.V., McGinnis R.S., Perkins N.C. (2020). Robust error-state Kalman filter for estimating IMU orientation. IEEE Sens. J..

[38] Vitali R.V., Perkins N.C. (2020). Determining anatomical frames via inertial motion capture: A survey of methods. J. Biomech..

